# The impact of respiratory tract mimicking media on antifungal susceptibility testing

**DOI:** 10.1128/aac.00225-25

**Published:** 2025-06-17

**Authors:** Roya Vahedi-Shahandashti, Isabella Hofer, Hollie Leighton, Joanne L Fothergill, Daniel R Neill, Cornelia Lass-Flörl

**Affiliations:** 1 Institute of Hygiene and Medical Microbiology, Medical University of Innsbruck 172142, Innsbruck, Austria; 2 Institute of Infection, Veterinary and Ecological Sciences, University of Liverpool 4591, , Liverpool, United Kingdom; 3 Division of Molecular Microbiology, School of Life Sciences, University of Dundee 98264, Dundee, United Kingdom; University Children's Hospital Münster, Münster, Germany

**Keywords:** antifungal susceptibility testing, antifungal resistance, invasive fungal infections, respiratory tract disease, broth microdilution

## Abstract

The rise in antifungal resistance and changing epidemiology underscores the need to improve antifungal susceptibility testing. This study tested the standard medium RPMI_1640_ and healthy lung and sinus media against *Aspergillus*, *Mucor*, and *Candida* species. *Candida* showed greater variability in minimum inhibitory concentrations (MICs) compared with molds, with higher MICs in respiratory tract-mimicking media than in RPMI_1640_. *Aspergillus terreus* was the most affected mold, showing higher MICs for certain antifungals in these media.

## INTRODUCTION

Invasive fungal infections (IFIs) have been rising in recent decades, primarily due to the growing population of immunosuppressed patients (1), which has led to increased reliance on systemic antifungal drugs, such as echinocandins, polyenes, and triazoles (2). However, the widespread use of these drugs has contributed to developing antifungal resistance, posing a significant challenge to treatment efficacy (3). Consequently, antifungal susceptibility testing (AFST) has become increasingly crucial for optimizing therapeutic strategies, guiding patient management, and monitoring antifungal resistance (4, 5). AFST is recommended in situations, such as confirmed or suspected IFIs, suspected cases of acquired resistance, and for patients experiencing refractory, relapsing, or breakthrough infections (6, 7). Several methods of AFST are currently used or under development (5). The main objective behind the standardization and periodic optimization of reference guidelines, such as those from the Clinical and Laboratory Standards Institute (CLSI) and the European Committee on Antimicrobial Susceptibility Testing (EUCAST), is to minimize the effect of various factors on AFST results, including inoculum size, incubation time, temperature, shaking conditions, and the composition of the medium (5, 8, 9). The growth medium is presumably one of the most profoundly influential factors of AFST results among the parameters noted, affecting the minimum inhibitory/effective concentration (MIC/MEC) value (8, 10, 11). The significance of host-mimicking environments in accurately reflecting pathogen behavior is widely recognized. However, RPMI_1640_, commonly used as a standard medium in reference AFST, may inadequately replicate the chemical and immunological complexity of the host environment, which underscores the need for further investigation into more representative testing media.

To address this gap, we evaluated the effect of culture medium on *in vitro* AFST by comparing artificial respiratory tract media, Healthy Sinus Medium (HSM) (12) and Healthy Lung Medium (HLM) (12), with standard RPMI_1640_ medium (13). The activity of frequently used representative antifungal agents from three major classes, polyene (amphotericin B; AmB, Sigma-Aldrich, A4888), azoles (voriconazole; VRC, Sigma-Aldrich, PZ0005; isavuconazole; ISA, Sigma-Aldrich, SML2357), and echinocandins (anidulafungin; ANF, Sigma-Aldrich, SML2288), commonly utilized in clinical diagnostic laboratories, was assessed against selected mold and yeast pathogens typically associated with infections of the lung and sinuses (14–17), including *Aspergillus fumigatus* (*n* = 3), *Aspergillus flavus* (*n* = 2), *Mucor* spp. (*n* = 2), *Aspergillus* niger (*n* = 2), *Aspergillus terreus sensu stricto* (s.s) (*n* = 8), *Aspergillus citrinoterreus* (*n* = 2), *Aspergillus alabamensis* (*n* = 2), *Aspergillus hortae* (*n* = 2), *Candida albicans* (*n* = 2), *Candida tropicalis* (*n* = 2), *Nakaseomyces glabratus* (formerly *Candida glabrata*) (*n* = 2), *Pichia kudriavzevii* (formerly *Candida krusei*) (*n* = 2) and *Kluyveromyces marxianus* (formerly *Candida kefyr*) (*n* = 1), according to the EUCAST guideline (13, 18). *A. fumigatus* (ATCC 204305), *A. flavus* (ATCC 204304), *C. krusei* (ATCC 6258), and *C. parapsilosis* (ATCC 22019) were used as control strains. The minimum inhibitory concentrations (MICs), defined as the concentration at which no hyphal growth was detected, were determined for amphotericin B and azoles. For echinocandins, the minimal effective concentrations (MECs), which represent the concentration at which hyphal growth was significantly altered and blunted colonies formed, were assessed. Susceptibility testing was performed in duplicate, with any significant variations in MIC or MEC defined as changes greater than two log₂ dilutions.

The MIC and MEC values for all tested mold and yeast species are presented in Tables 1 and 2, respectively. Among the mold species, most *A. terreus* s.s. isolates and one *A. hortae* isolate predominantly showed higher MICs (more than two log2 dilutions), particularly for AmB and occasionally ISA, in HSM and HLM media compared with RPMI_1640_ (Table 1). In comparison, among the yeast species, a broader range of *Candida* species—including *C. albicans*, *C. tropicalis*, *P. kudriavzevii* (formerly *Candida krusei*), and *K. marxianus* (*Candida kefyr*)—exhibited higher MIC values in respiratory-mimicking media than in RPMI_1640_, primarily for azoles (Table 2). Positive growth controls indicated that each of the three media tested successfully supported fungal growth at the designated reading times. Overall, tested *Aspergillus* species were generally less influenced by the tested media compared to *Candida* species, with results indicating more variability among individual strains rather than a consistent response pattern. Selecting the appropriate test medium that supports fungal growth without interfering with drug activity is essential (5), as fungal isolates grow at different rates on various media, leading to variable MIC values (5, 19). It has been demonstrated by some studies (20–22) that the susceptibility of fungi depends on the growth stage, with some indicating increased susceptibility in the exponential phase compared to the lag phase; this may explain how nutrient-limited media can lead to a prolonged lag phase for the organism and consequently affect the potential efficacy of the applied antifungal, underscoring the importance of the testing medium’s impact on AFST results. While no single culture medium can support the growth of all clinically relevant fungi, RPMI_1640_, a fully defined synthetic formula with minimal batch variation, was selected as the standard medium for AFST due to its high interlaboratory agreement, specifically for yeast (23, 24), although there was no evidence that this medium was suitable for filamentous fungi (25). However, certain disadvantages raise concerns about RPMI_1640_ as a suitable medium for all fungi and antifungals, including its nutrient-limited composition, which can impact fungal growth (19), and its inability in some cases to differentiate between amphotericin B-resistant or tolerant isolates and susceptible ones (9, 11, 26). Therefore, the opinion that RPMI_1640_ medium is universally suitable for all fungal species and antifungals warrants careful reconsideration, and consistent results for quality control strains should not be considered as definitive evidence of its effectiveness for all fungi and antifungals. *In vitro* drug susceptibility tests are usually performed under artificial growth conditions that supply the organism with readily metabolized carbon, such as glucose, and nitrogen, predominately glutamine and arginine, usually at non-physiological levels (12). Such conditions are known to inhibit the expression of virulence-associated genes *in vitro* (27).

**TABLE 1:** Susceptibility profiles of amphotericin B (AmB), voriconazole (VRC), isavuconazole (ISA), and anidulafungin (ANF) against some mold representatives species in three different media; RPMI_1640_, Healthy Sinus Medium (HSM), and Healthy Lung Medium (HLM), according to the EUCAST method^*b*^

Species	Antifungals	MIC-MEC [mg/L]-Media			
		RPMI_1640_	HSM	HLM	Susceptibility categorization
*A. fumigatus* 1	AmB VRC ISA ANF	1 8 >8 0.007	1 >8 >8 0.015	2 >8 >8 0.015	



*A. fumigatus* 2	AmB VRC ISA ANF	2 8 >8 0.007	1 >8 >8 0.015	2 8 >8 0.031	



*A. fumigatus* 3	AmB VRC ISA ANF	2 1 1 0.007	1 2 1 0.015	2 0.5 1 0.031	



*A. flavus* 1	AmB VRC ISA ANF	8 2 4 0.007	8 2 2 0.015	4 2 2 0.015	



*A. flavus* 2	AmB VRC ISA ANF	2 1 1 0.015	1 2 2 0.031	2 1 2 0.015	



*Mucor* spp. 1	AmB VRC ISA ANF	0.25 –^*a*^ >8 –	0.125 – >8 –	1 – >8 –	



*Mucor* spp. 2	AmB VRC ISA ANF	0.5 – >8 –	0.5 – >8 –	0.25 – >8 –	



*A. niger* 1	AmB VRC ISA ANF	1 2 8 0.015	2 4 8 0.007	4 4 8 0.015	



*A. niger* 2	AmB VRC ISA ANF	1 1 2 0.031	1 2 4 0.007	1 2 4 0.007	



*A. terreus* s.s 1	AmB VRC ISA ANF	**2** 4 **2** 0.007	**>8** 8 **>8** 0.015	**>8** 8 **8** 0.031	Wt to Non-Wt



*A. terreus* s.s 2	AmB VRC ISA ANF	**1** 4 2 0.003	**4** 8 8 0.007	**>8** 8 8 0.015	Wt to Non-Wt



*A. terreus* s.s 3	AmB VRC ISA ANF	**2** 4 2 0.003	**>8** 8 4 0.015	**>8** 8 8 0.015	Wt to Non-Wt
*A. terreus* s.s 82	AmB VRC ISA ANF	>8 2 2 0.007	>8 4 8 0.015	>8 4 4 0.015	
*A. terreus* s.s 192	AmB VRC ISA ANF	>8 1 **1** 0.007	>8 4 **8** 0.015	>8 2 4 0.015	

					S to R

*A. terreus* s.s 9	AmB VRC ISA ANF	>8 1 1 0.007	>8 2 4 0.015	>8 2 4 0.015	
*A. terreus* s.s S2	AmB VRC ISA ANF	1 0.5 1 0.007	1 1 2 0.015	0.5 1 2 0.015	
*A. terreus* s.s S3	AmB VRC ISA ANF	0.5 0.5 1 0.007	1 1 2 0.015	0.5 1 2 0.015	
*A. citrinoterreus* 27	AmB VRC ISA ANF	>8 0.5 0.5 0.007	>8 1 2 0.015	>8 2 1 0.007	
*A. alabamensis* 63	AmB VRC ISA ANF	8 1 1 0.007	>8 2 4 0.015	>8 2 4 0.015	
*A. hortae* 119	AmB VRC ISA ANF	8 1 **0.5** 0.007	>8 2 **4** 0.007	>8 2 2 0.015	
*A. hortae* 142	AmB VRC ISA ANF	>8 0.5 1 0.007	>8 2 4 0.015	>8 2 4 0.015	
*A. citrinoterreus* 220	AmB VRC ISA ANF	>8 2 2 0.007	>8 2 4 0.015	>8 2 4 0.031	
*A. alabamensis* 225	AmB VRC ISA ANF	>8 2 2 0.003	>8 2 2 0.007	>8 2 2 0.015	
*A. fumigatus* (ATCC 204305)	AmB VRC ISA ANF	2 1 0.5 0.015	1 1 1 0.015	2 2 1 0.015	
*A. flavus* (ATCC 204304)	AmB VRC ISA ANF	2 2 1 0.007	2 2 2 0.031	4 4 2 0.031	

^*a*^ This symbol indicates that either the antifungal is not recommended for the specified fungal species or the medium is not applicable for that fungus, as it is not a known pathogen of the lung or sinus.

^*b*^ Significant changes are highlighted in bold. For strains and antifungal agents with defined clinical breakpoints or epidemiological cutoff values (ECOFF) according to EUCAST (28), susceptibility transitions across media are labeled as susceptible/resistant (S/R) or wild-type/non-wild-type (Wt/non-Wt).

**TABLE 2:** Susceptibility profiles of amphotericin B (AmB), voriconazole (VRC), isavuconazole (ISA), and anidulafungin (ANF) against some yeast representatives species in three different media: RPMI_1640_, Healthy Sinus Medium (HSM), and Healthy Lung Medium (HLM), according to the EUCAST method^*b*^

Species	Antifungals	MIC [mg/L] -Media			
		RPMI_1640_	HSM	HLM	Susceptibility categorization
*C. albicans* 1	AmB VRC ISA ANF	0.5 **0.008** **0.008** 0.008	0.25 **>4** **>4** 0.015	0.25 **>4** **>4** 0.015	
					S to R


*C. albicans* 2	AmB VRC ISA ANF	0.5 **1** 0.015 0.015	0.25 **>4** 0.125 0.008	0.25 **>4** 0.125 0.008	



*C. tropicalis* 1	AmB VRC ISA ANF	0.5 **0.06** 0.008 0.015	0.5 **2** 0.06 0.007	0.5 0.25 0.03 0.007	
					S to R


*C. tropicalis* 2	AmB VRC ISA ANF	**0.5** 4 **0.008** 0.03	**>4** >4 **0.06** 0.007	1 >4 **>4** 0.007	S to R

					S to R

*N. glabratus* 1 (formerly *Candida glabrata*)	AmB VRC ISA ANF	0.5 0.125 0.015 0.015	–^*a*^ – – –	0.5 0.125 0.06 0.03	
*N. glabratus* 2 (formerly *Candida glabrata*)	AmB VRC ISA ANF	0.5 0.06 0.03 0.008	– – – –	1 0.125 0.06 0.03	
*P. kudriavzevii* 1 (formerly *Candida krusei*)	AmB VRC ISA ANF	**1** **0.5** **0.25** **0.03**	**>4** **>4** **>4** **>4**	**>4** **>4** **>4** **>4**	S to R
					Wt to Non-Wt

					S to R
*P. kudriavzevii* 2 (formerly *Candida krusei*)	AmB VRC ISA ANF	1 0.25 **0.008** 0.03	1 0.5 **0.5** 0.06	4 0.5 **0.5** 0.06	
*K. marxianus* 21146779 (*Candida kefyr*)	AmB VRC ISA ANF	1 **0.015** **0.015** 0.015	0.5 **0.125** **0.25** 0.06	– – – –	
*C. parapsilosis* (ATCC 22019)	AmB VRC ISA ANF	1 0.03 0.008 1	0.5 0.125 0.06 0.5	1 0.06 0.03 1	
*C. krusei* (ATCC 6258)	AmB VRC ISA ANF	1 0.25 0.008 0.03	0.5 1 0.5 0.03	1 0.5 0.5 0.03	

^*a*^ This symbol indicates that either the antifungal is not recommended for the specified fungal species or the medium is not applicable for that fungus, as it is not a known pathogen of the lung or sinus.

^*b*^ Significant changes are highlighted in bold. For strains and antifungal agents with defined clinical breakpoints or epidemiological cutoff values (ECOFF) according to EUCAST (28), susceptibility transitions across media are labeled as susceptible/resistant (S/R) or wild-type/non-wild-type (Wt/non-Wt).

To address these limitations, HSM and HLM were examined to more accurately resemble the nutrient composition and physiological environment of the healthy human respiratory tract. The choice of “healthy” conditions is intended to establish a controlled, consistent, and standardized baseline that reflects normal host–pathogen interactions, in the absence of inflammation or immunosuppression, which can vary between individuals. While both HSM and HLM media incorporate host-like carbon and nitrogen sources, the sinus medium is more nutritionally limited, particularly in carbon and nitrogen availability (12). Both media also contain ionic profiles, protein concentrations, host antimicrobial peptides and enzymes, mucin, and extracellular DNA, components that are absent in RPMI_1640_ (12). These physiologically relevant conditions can influence fungal metabolism and growth dynamics, potentially impacting AFST, particularly for agents whose activity is modulated by the fungal metabolic state and membrane composition (19, 20, 29). Further investigation is warranted to assess the growth kinetics of representative mold and yeast isolates in airway-mimicking media compared with RPMI_1640_, to clarify the potential influence of growth stages and metabolic state across media on antifungal efficacy. With further validation, HSM and HLM may serve as more physiologically relevant and standardized alternatives to RPMI_1640_ for AFST, helping to bridge the gap between overly simplistic *in vitro* conditions and the complex *in vivo* airway environment, particularly for fungal pathogens associated with the sinuses and lungs.

## Data Availability

The authors confirm that all data/protocols are detailed within the article.
